# Language Matters: What Not to Say to Patients with Long COVID, Myalgic Encephalomyelitis/Chronic Fatigue Syndrome, and Other Complex Chronic Disorders

**DOI:** 10.3390/ijerph22020275

**Published:** 2025-02-13

**Authors:** Nancy J. Smyth, Svetlana Blitshteyn

**Affiliations:** 1 School of Social Work, University at Buffalo, Buffalo, NY 14421, USA; 2 Department of Neurology, Jacobs School of Medicine and Biomedical Sciences, University at Buffalo, Buffalo, NY 14203, USA; sb25@buffalo.edu; 3 Dysautonomia Clinic, Williamsville, NY 14221, USA

**Keywords:** Long COVID, ME/CFS, dysautonomia, patient–practitioner communication, physician–patient communication, communication skills, patient engagement, complex disorders, chronic disorders

## Abstract

People with Long COVID, myalgic encephalomyelitis/chronic fatigue syndrome (ME/CFS), and other complex chronic disorders consistently report having difficulty obtaining effective and compassionate medical care and being disbelieved, judged, gaslighted, and even dismissed by healthcare professionals. We believe that these adversarial interactions and language are more likely to arise when healthcare professionals are confronting complex chronic illnesses without proper training, diagnostic biomarkers, or FDA-approved therapies. These problematic conversations between practitioners and patients often involve specific words and phrases—termed the “never-words”—can leave patients in significant emotional distress and negatively impact the clinician–patient relationship and recovery. Seeking to prevent these destructive interactions, we review key literature on best practices for difficult clinical conversations and discuss the application of these practices for people with Long COVID, ME/CFS, dysautonomia, and other complex chronic disorders. We provide recommendations for alternative, preferred phrasing to the never-words, which can enhance therapeutic relationship and chronic illness patient care via compassionate, encouraging, and non-judgmental language.

## 1. Introduction

Clinical care and communication surrounding complex chronic disorders, such as Long COVID, myalgic encephalomyelitis/chronic fatigue syndrome (ME/CFS), dysautonomia, and other disorders involving chronic fatigue and chronic pain has been a challenging task, influenced by a lack of clinical training, controversy, internalized biases, and ableism. Although there is strong evidence of abnormal pathophysiology underlying these conditions [1], biopsychosocial explanation and psychogenic etiologies continue to influence how medical professionals relate to and communicate with patients affected by these disorders: paternalistic relationship and false attribution of physical disorders to disturbances of the mind and psyche are among some of the major barriers to effective therapeutic relationship and treatment of patients with these disorders [2,3].

Both Long COVID and ME/CFS are complex, with Long COVID being a relatively new diagnostic entity and ME/CFS, despite its longer history, still not taught in medical training, making both disorders vulnerable to stigmatization and trivialization [2,3,4]. Both disorders have been characterized as complex, multisystemic, and extensively psychologized, with patients encountering substandard medical care, disbelief, and stigma [3]. This highlights a structural barrier that is unique to ME/CFS, Long COVID, and other complex chronic illnesses that remain under-investigated: limited research funding and a lack of education and medical training are major contributors to these conditions being labeled as “invisible illnesses” [4]. The multisystemic nature and complexity of these conditions may lead to patients and their healthcare professionals being squarely at odds with their perspectives on these conditions; healthcare professionals assert one version of reality (“you don’t look sick”; “your tests are normal”), and patients are frustrated with their experience (profound exhaustion and inability to engage in physical and cognitive tasks) being disbelieved and denied the appropriate medical care, categorizing these interactions as “gaslighting” [5]. We believe that these adversarial relationships and language are more likely to arise when healthcare professionals are confronting complex chronic illnesses without proper training, diagnostic biomarkers, or FDA-approved therapies. The problematic conversations that transpire between clinicians and patients often involve specific words and phrases—termed the “never-words [6]”—that can leave patients feeling upset, angry, fearful, confused, and demoralized. Situations where patients are blamed for their own illness (e.g., “you feel sick because you are out of shape”) or told to think positively (e.g., “you need to stop thinking about your symptoms so much”) when they have significant post-acute infectious cardiovascular, pulmonary, neurologic, and immunologic complications, or when these arise in the context of systemic disease, create significant distress and distrust for patients and undermine therapeutic relationships, disease management, and chances for improvement or recovery. Seeking to prevent these destructive clinical interactions, we will review key literature on best practices for difficult clinical conversations and then discuss the application of these practices for people with Long COVID, ME/CFS, dysautonomia, and other complex, chronic, and disabling disorders. We will provide recommendations for alternative, preferred phrasing to the never-words, which can enhance therapeutic relationship and chronic illness patient care via compassionate, encouraging, and non-judgmental language.

## 2. What Are Long COVID, ME/CFS and Dysautonomia

Long COVID, ME/CFS, and dysautonomia are complex chronic conditions that often involve challenging clinical conversations and interactions between patients and healthcare professionals. Understanding these disorders is key to understanding the challenges that can arise in these dialogs.

According to the National Academy of Sciences, Engineering, and Medicine definition, Long COVID is defined as “an infection-associated chronic condition that occurs after SARS-CoV-2 infection and is present for at least 3 months as a continuous, relapsing and remitting, or progressive disease state that affects one or more organ systems [3]”. It can follow asymptomatic, mild, or severe SARS-CoV-2 infection [3]. The condition may range from mild to severe, with patient complaints such as fatigue, exercise intolerance, post-exertional malaise, cognitive impairment (often referred to as “brain fog”), diffuse chronic pain, sleep disruption, autonomic dysfunction, including POTS, migraines, gastrointestinal symptoms, and many others. The onset of symptoms may be continuous from the time of infection or delayed in onset by weeks or months following an apparent full recovery from the acute phase of infection [3]. Disability related to Long COVID may result in profound functional impairments in self-care, as well as family, social, school, and occupational roles. Fifty percent of patients with Long COVID qualify for the diagnosis of ME/CFS [7], which similarly results in many of the same themes, biases, and controversies historically characterizing ME/CFS.

Unlike Long COVID, ME/CFS has existed for centuries and has been rife with controversy, politicization, and patient mistreatment. In a report published by the National Academy of Medicine (previously the Institute of Medicine) in 2015, the diagnostic criteria for ME/CFS in adults and children have been defined. Three required symptoms and at least one of two additional symptoms were required to be present for the diagnosis of ME/CFS, including a substantial reduction or impairment in the ability to engage in pre-illness levels of activity (occupational, educational, social, or personal life) lasting for more than 6 months accompanied by profound fatigue, which is not alleviated by rest. Post-exertional malaise (PEM) and unrefreshed sleep must be present [2,8]. Either cognitive impairment or orthostatic intolerance are also required for the diagnosis [2].

Dysautonomia is an umbrella term that refers to any disturbance or dysfunction of the autonomic nervous system and is a major pathophysiologic mechanism of Long COVID and ME/CFS. Autonomic disorders, such as postural orthostatic tachycardia syndrome (POTS), neurocardiogenic syncope, and orthostatic hypotension, may follow SARS-CoV-2 and other infections and are common comorbidities of ME/CFS, autoimmune and inflammatory conditions, and hypermobility spectrum disorders [9,10,11]. Other complex chronic disorders that have been historically poorly understood or neglected by the medical community include fibromyalgia, chronic pain syndromes, undifferentiated connective tissue disorders, mast cell activation syndrome, hypermobility spectrum disorders, autoimmune disorders that do not fit into the currently accepted diagnostic labels (Sjogren’s syndrome, antiphospholipid syndrome and others), and genetic conditions, including mitochondrial disorders, that to date, have not been defined.

Healthcare professionals are often unfamiliar with the recognition, diagnosis, and treatment of these disorders [2,3,4,5,9]. Funding has recently been provided for Long COVID healthcare professional education to increase awareness and training among physicians and allied health practitioners [12].

## 3. Review: Healthcare Communication Skills for Serious Illness

Effective communication between healthcare professionals and patients has long been recognized as essential to providing quality healthcare—the quality of the patient–clinician relationship is strongly predictive of positive patient care outcomes [13,14,15]. However, it can be challenging to keep this front and center in the busy day-to-day healthcare setting, especially when providing care for patients with complex chronic disorders. Patients facing difficult medical situations, such as life-altering chronic medical conditions, feel especially scared, vulnerable, and out of control, which adds a level of intensity and emotional charge to these interactions. In these emotionally charged situations, practitioners may feel deskilled and revert to more authoritative language and styles of communication, which can then leave patients feeling discounted and unheard, evoking fear and distrust. The result is that communication breaks down, creating barriers to patient care. Fortunately, the literature on communicating during cancer care, serious illness, and end-of-life care offers guidance that can be extended to inform practitioner-patient communications in other serious, complex illnesses. Unfortunately, there is little training or guidance on how to adapt these communication skills to communication about chronic, disabling, and life-altering chronic medical conditions such as neurologic and autoimmune conditions.

There are several well-validated models for effective practitioner–patient communication conversations involving serious illness. The Critical Care Communication (C3) project [16] applies the widely used VitalTalk skills package [17,18], an extensively researched program [19] with four core skills (“Ask-Tell-Ask”, recognize/respond to feelings, ask permission to move the conversation forward, and express curiosity “tell me more”) [17]. C3 focuses on giving bad news, achieving shared treatment goals, and exploring the limitations of life-sustaining care. CLEAR conversations (Connect, Listen, Empathize, Align, Respect) [20] is embodied in an app to facilitate the transfer of bundled simulation communication training (role play simulations with actors) and structured feedback to real-world Intensive Care Unit family meetings—the app was created to help practitioners apply skills in this high-stress environment. The REDE model (Relationship, Establishment, Development, Engagement) [21] is more broadly focused on general healthcare practice, not just serious medical illness, using guided practice and coaching for communication across the REDE stages. Finally, the SICG (Serious Illness Conversation Guide) [22,23] focuses on end-of-life conversations that probe patients’ understanding, information preferences, goals, fears/worries, and important functioning/abilities in addition to other key aspects related to end-of-life planning with oncology patients.

Common to these communication training programs is (a) relationship building (through questions, listening, conveying empathy), (b) sharing information briefly first, with a headline, (c) questions to gather the patient’s perspective and values, and (d) collaboration for care planning. Importantly, most of these programs are adapted to reflect the concerns and issues unique to a particular clinical focus, such as Intensive Care (C3 and CLEAR), end-of-life and palliative conversations, as well as oncology (SICG). This makes sense because we communicate about specific content, i.e., “if you become sicker, how much are you willing to go through for the possibility of gaining more time [22]?” or surgeons being trained to present stories to highlight the best- and worst-case surgery outcome scenarios [24]. It is clear from the literature that incorporating unique aspects of specialized healthcare practice can assist in applying effective communications skills to these specific healthcare contexts.

## 4. Structural Barriers to Effective Communication with Complex Chronic Conditions

Lee and colleagues [6] highlighted that the treatment of serious illness can be stressful for both the practitioner and the patient. Specifically, they noted that the emotionally charged nature of these conversations might cause practitioners to rely on phrases and words that are poorly matched to the patients’ circumstances at a time when patients are feeling especially vulnerable, stressed, and listening closely to every word. They observed that there also are structural barriers that can derail effective communication beginning with the power imbalance between practitioner and patient. Medical practitioners have extensive knowledge and expertise, and yet while patients are experts on their own lives and values, they enter the relationship feeling vulnerable—due to illness—which can make it harder for them to articulate and organize their thoughts, especially in a time-limited interaction. These interactions also occur in a healthcare context where clinician-patient interaction time is limited, and within hospitals, where it can be unclear who in the team should take the lead to ensure that essential conversations occur with a patient. Adding to this list, we note that twenty to fifty percent of a significant subset of primary care patients report experiences of abuse in childhood [25,26] which can make them mistrustful of healthcare professionals in adulthood, including within healthcare settings [27]. Skills in relationship building, exploring concerns, and collaborating on care provide a good start to overcoming these challenges.

It is because of this need to adapt communications to the unique issues with serious illness that Lee and colleagues identified the importance of never-words. Based on this model, and, consistent with the literature reviewed earlier, we propose that the never-words for patients need to be matched to the unique experience of patients with ME/CFS, Long COVID and/or other chronic disabling conditions. While serious illness of all types evokes fear and powerlessness, these emotions will be amplified with complex chronic disorders that are broadly defined, poorly understood mechanistically, and lack clinically available diagnostic biomarkers and FDA-approved therapies.

Finally, even when clinicians and patients are in agreement about a chronic complex condition, because of the complexity and chronicity of these illnesses, it is easy for healthcare professionals to feel inadequate and ineffective in their professional capacity due to a lack of FDA-approved therapies for patients with Long COVID, ME/CFS, and other complex chronic disorders. These feelings can result in clinicians offering pat phrases or simply telling the patient their conditions are not treatable. Those addressing these communication barriers also have identified that humility, that is, specifically acknowledging what you do not know, builds trust [4,28], and recent research on science communication confirms this, as well [29].

As a result, we are extending the important work of Lee et al. [6] by presenting never-words for healthcare professionals to avoid using in communications with patients who have Long COVID, ME/CFS, dysautonomia, and other complex chronic disorders, explaining their likely impact, and suggesting alternatives. The first author has several decades of experience as a clinician with patient engagement, teaching patients how to communicate about invisible illnesses, and, more recently, helping people with Long COVID to communicate and advocate with healthcare professionals and employers. The second author has extensive experience as a clinician specializing in complex chronic disorders, including Long COVID, dysautonomia, and ME/CFS. Given our perspective as healthcare professionals, it is important to note that this affects how we view this subject matter, which could lead to bias about never-words. For this reason, we enlisted feedback from a patient advocate related to the never-words and have taken their feedback into consideration.

In Table 1, we identify the never-words, discuss their impact, and suggest alternate phrasing, which can enhance therapeutic relationship and patient care with compassionate, encouraging and non-judgmental language.

We chose to include these particular situations to ensure we captured the breadth of the types of negative interactions reported by patients [5,30,31,32]. It also provided us with an opportunity to educate healthcare professionals about the range of impacts these statements can have. Finally, we note that some of never-words highlight the role that gender bias can play in these challenging dialogs [5,30,33], which is not surprising given that both Long COVID and ME/CFS are more prevalent in women [2,3,34].

## 5. Conclusions

Communicating with patients about complex, chronic, and disabling illnesses can be an uncomfortable, nuanced, or highly emotional interaction that few healthcare professionals have been taught to engage in throughout their medical training. Clinicians may be apprehensive while approaching the diagnostic and therapeutic management of these complex chronic illnesses without proper training, diagnostic biomarkers, or FDA-approved therapies. Patients are likely to enter these relationships having already encountered multiple obstacles to obtaining effective and compassionate medical care, including dismissal, denial, misdiagnosis with psychiatric disorders, personalized biases, and medical neglect [5]. In addition, patients with Long COVID, ME/CFS, and other chronic disorders have experienced significant losses because of these disorders, and these losses have a negative impact on them and on their families. For these reasons, relationship-building skills, such as asking questions about their experiences prior to seeking care with you and conveying empathy, as well as avoiding never-words, will all be important to developing a strong therapeutic relationship.

While effective healthcare professional-patient communication should follow well-established structures and processes for communicating about serious illness, it should also avoid never-words that have special meaning within this unique healthcare situation—these never-words may be detrimental to effective communication and serve as significant barriers to effective clinical care and therapeutic relationships.

## Figures and Tables

**Table 1:** Never-words, their impact, and suggested alternatives.

Never-Words	Explanation and Impact	Alternative
“You don’t look sick”.	Many patients may appear healthy, but feel very sick with various symptoms, including fatigue and pain.	Please refrain from commenting on their appearance.
“You need to stay positive”.	Saying this to patients with debilitating symptoms with limited treatment implies that the patient did not stay positive or that the patient’s attitude is to blame for feeling or staying sick.	“I know it can feel discouraging to feel so sick, and especially for so long. We will work on this together”.
“At least it’s not cancer”.	Minimizing symptoms and disabilities is not well-received by patients who are suffering with non-terminal, but debilitating, and disabling conditions.	Comparing diseases to make a patient feel better is a strategy that is best avoided, since it usually has the opposite impact.
“Learn to live with this”.	While this may be practical advice, many patients have already adjusted to living with their illness, but they want to live better and be more functional.	“I know this illness can really disrupt your life. What did you do in order to adjust to this?”
“Good news: Your tests are all normal”.	This is good news for medical professionals, but patients may not care about the numbers or test results if they feel sick. This may also imply to patients that because their tests are normal, they have no reason to feel sick.	The tests we have run so far are not showing any abnormalities, and the good news is that we have excluded certain conditions based on the results of these tests.
“Many people have it worse”.	Deflecting the patient’s suffering can be perceived as gaslighting by the sufferer.	Please refrain from comparing patient’s diseases and experiences.
“Have you tried___(lifestyle measures: yoga, going for a walk, diet, etc.?)”	Many patients have already tried various lifestyle measures without benefits and are seeking further treatment from healthcare professionals, not recommendations of the same lifestyle measures.	What are the things you have tried that have or have not helped you?
“You feel sick because you are____(psychological label: anxious, depressed, stressed)	Many patients with chronic illness do have comorbid depression, anxiety, PTSD, and other psychiatric disorders, but in many patients, it is not an explanation nor a justification for why they feel ill. Further, it is important to note that people living with a chronic complex condition experience many losses due to having that condition.	If you are suspecting significant psychological or psychiatric comorbidities, please refer your patient to a mental health professional to address these issues.
“You feel sick because you are____(fitness label: deconditioned, overweight, underweight, out of shape)	Many patients have been previously healthy and active, and many patients want to restart exercising and lead an active lifestyle but cannot due to fatigue, pain, and post-exertional malaise.	Please refrain from commenting on the patient’s fitness level or body habits. A referral to a physical therapist with expertise in chronic fatigue may be helpful.
“You feel sick because you are____(hormonal status: perimenopausal, menopausal, postmenopausal, postpartum, pregnant, menstruating, ovulating)	Many patients with Long COVID, MECFS, and other chronic disorders are women who can often differentiate between hormonal symptoms and symptoms of chronic disease. Additionally, hormonal influence on symptoms is well-documented but is not an explanation or the cause of the underlying disease.	Please refrain from commenting on the patient’s hormonal status. A referral to a gynecologist or endocrinologist might be appropriate if there are concerns of hormonal abnormalities or need for hormonal supplementation.
“You need to____(instruction as cures: lose/gain weight, start exercising, get fresh air, get out of the house/bed, get a job, get a hobby, start dating etc.)”	While a healthy lifestyle is important, the patient did not choose to stop it: the lifestyle changed as a result of the illness. Additionally, while lifestyle measures are important, they are unlikely to cure or effectively treat the underlying medical condition.	“When you feel better, we will work together toward a common goal of improved quality of life and a healthier lifestyle”.
“You look too___(appearances: good, young, skinny, pretty)__to be sick”.	Comments on appearances are inappropriate because patients with chronic illness may not look sick like patients with acute illness. Many actually hide their ill-appearing looks, especially when seeing a healthcare professional.	Please refrain from commenting on patient’s appearance.
“We don’t have any treatment for your illness”.	While this may be true for some illnesses, given no FDA-approved therapies, symptomatic treatment is available, and the patient should not be made to feel like they are being abandoned by the medical team.	“We will talk about the available treatments we have that can make you feel better”.
“You need to stop thinking about your symptoms so much”.	In our experience, improved symptom control results in many patients improving their function and decreasing the negative thoughts and feelings about their symptoms. In those patients who continue to perseverate about their symptoms, psychological support, and cognitive-behavioral therapy may be appropriate.	“You have good awareness of your symptoms. I’m wondering if we can come up with a way for you to easily track them, so we that we can see the small changes when you begin to feel better”.
“You have to find something productive to do with your time”.	This statement assumes that patients are bored or have too much time on their hands, whereas, for most patients, having complex chronic illnesses is time- and energy-consuming and may be equivalent to having a full-time job managing disease and medical care. Additionally, many patients are not physically and/or cognitively well enough to be productive.	“Try to distract yourself with doing pleasurable and meaningful things that you can still do for short periods of time”.
“Don’t confuse your Google search with my medical degree”.	This statement has become popular among healthcare professionals, given various online information platforms and social media groups that patients use to obtain medical information. However, we find that many patients with complex chronic illnesses had to become educated in their disorder out of necessity, given limited help from medical professionals.	“I am glad you’re reading about your illness and educating yourself on possible tests and treatments. Thank you for bringing this information to me. I will look through it and let you know my thoughts”.

## Data Availability

No data was used in this paper.
